# The value and promise of embedded research

**DOI:** 10.1186/s12961-021-00744-8

**Published:** 2021-08-11

**Authors:** Abdul Ghaffar, Anuradha Gupta, Aboubacar Kampo, Soumya Swaminathan

**Affiliations:** 1 Alliance for Health Policy and Systems Research, WHO, Geneva, Switzerland; 2 Gavi, The Vaccine Alliance, Geneva, Switzerland; 3 UNICEF, New York, United States of America; 4 WHO, Geneva, Switzerland

**Keywords:** Implementation, Embedded approach, Decision-maker led research

Despite a growing realization of the added value of the use of evidence to inform decision-making in low-and-middle-income countries (LMICs), several barriers to its uptake and use persist. These include inadequate contextually sensitive research, lack of alignment to policy cycles, and limited capacities among policymakers to appraise and use evidence. Many of these barriers are exacerbated by the inadequate involvement of decision-makers at various levels in establishing research priorities and generating research evidence [1].

Existing research uptake mechanisms, developed in the context of clinical practice and medical guidelines, largely focus on the packaging of evidence for ease of understanding for front-line practitioners. However, the packaging of evidence alone is an inadequate approach to enhance evidence use in policy development and programme implementation [2]. Given the central role of context in determining both what evidence is needed and how this evidence can be applied to the problem at hand, enhancing evidence use in policies and programmes requires an approach that puts decision-makers at the centre of the evidence generation process.

In this context, the WHO Strategy on Health Policy and Systems Research recommended the embedding of research, with a view to catalysing evidence-informed decision-making. The embedded approach is one where decision-makers and researchers collaborate on prioritisation, conduct and translation of research [3]. It is envisioned that this approach would lead to a system where ‘researchers and policymakers were linked, and in which the need for evidence to inform policy was understood by decision-makers’ [4].

One specific way to enable the embedded approach is having decision-makers lead research, giving them greater ownership within the research process. This is of particular relevance in the area of immunisation, where embedded research may help solve issues such as when scale-up has stalled or particular populations are under-served due to systemic and structural barriers to access. Addressing these issues often involves overcoming context specific challenges that are best known to and understood by the decision-makers involved closely with the programmes [5]. Their involvement in the research thus has the potential to improve the understanding of the problem and enhance the development of relevant solutions, increasing the reach and effectiveness of immunisation programmes.

In light of this, the Alliance for Health Policy and Systems Research, WHO in collaboration with UNICEF and Gavi, the Vaccine Alliance launched two research calls (in 2015 and 2016), focusing on priority issues around immunisation, termed Decision-Maker Led Implementation Research on Immunization (DELIR). Notably, project teams were led by decision-makers, something that was a requirement to receive funding and was intrinsic to the programme’s design.

A full description of the DELIR initiative is provided in the editorial published in *Health Research Policy and Systems* [6]. To summarise, 14 projects across ten countries (Chad, the Democratic Republic of the Congo, Ethiopia, India, Kenya, Nigeria, Pakistan, Somalia, Uganda and Viet Nam) explored questions related to a variety of issues around vaccine and immunisation coverage, demand, programme management and programme delivery.

Many tangible examples from DELIR demonstrate the value of embedded research in increasing evidence uptake into policy and practice.

For example, in Chad, to address context-specific challenges for vaccine delivery for hard-to-reach populations, specifically nomadic communities in Dnamadji District, a demand-creation communications strategy for the ‘One Health’ vaccination programme was developed by the research team in collaboration with community members. Post dissemination, the team worked to integrate this strategy within the immunisation programme [5]. There was also a perceived increase in national efforts to engage nomadic communities. Teams from other countries also reported that the embedded approach was beneficial for producing contextually meaningful evidence [7].

In another example, in Nigeria, the team developed policy guidance on the use of participatory action research in social mobilisation to address bottlenecks in service delivery and improve immunisation coverage in Ogun State [5]. Subsequent changes included an increase in immunisation coverage, strengthened capacity of the community in participatory action approaches, the continued used of participatory action research specifically in polio outbreaks and geographic scale-up of the strategy [7, 8].

Not only has this programme of research improved vaccination outcomes and influenced policy and practice, but it has also changed mindsets particularly with respect to the role of different stakeholders at each stage of the research process. Participants in DELIR have cited benefits such as increased research capacity, enhanced understanding of the value of evidence-informed decision-making and in the critical analysis of programming challenges, strengthened relationships between researchers, implementers and decision-makers, and the reconfiguration of power relations. It has also facilitated the identification of research questions that are of relevance to marginalised and vulnerable communities, thereby enhancing equity [7]. DELIR has enabled decision-makers to move from playing a relatively passive role as the recipients of research products to active co-producers of new knowledge. This process greatly strengthens the value of the evidence produced for day-to-day decision-making.

While the DELIR initiative has demonstrated the value of embedded research to improve the uptake and use of evidence leading to improvements in health systems, policies and programmes, the further adoption of the embedded approach requires galvanising action among funders at national and global levels. To ensure adequate funding, we recommend that major global health funders and national governments earmark funds for contextually sensitive research carried out in collaboration with policymakers within programme budgets. This would be a first step to making the generation of such evidence a required part of programmatic activities. In addition to this, funders need to catalyse building and sustaining processes and institutional arrangements within ministries of health to encourage the use of research as an intrinsic part of decision-making. This includes providing opportunities for ongoing engagement through interaction and dialogue between researchers and decision-makers to identify areas where evidence is needed, mandating the consideration of evidence in the development of new policies and programmes, as well as providing training to decision-makers at each level of the health system in appraising and using evidence as part of their day to day work [4]. Taken together, these measures can play a major role in contributing to a culture of evidence informed decision-making that is critical to successfully move towards universal health coverage (UHC) and the Sustainable Development Goals.

## Data Availability

Not applicable. The manuscript does not contain any data.
